# Pheasant Egg White Lysozyme vs. Hen Egg White Lysozyme: A Comparative Study of Enzymatic Activity

**DOI:** 10.3390/foods15193402

**Published:** 2026-09-24

**Authors:** Liliya I. Mukhametova, Dmitry O. Zherdev, Anton N. Kuznetsov, Julia V. Burykina, Olga N. Yudina, Svetlana M. Filimonova, Sergei A. Eremin, Vadim B. Krylov, Nikolay E. Nifantiev

**Affiliations:** 1 Faculty of Chemistry, M.V. Lomonosov Moscow State University, Leninsky Gory 1/3, 119991 Moscow, Russiame@zherderini.ru (D.O.Z.);; 2 Laboratory of Glycoconjugate Chemistry, N.D. Zelinsky Institute of Organic Chemistry, Russian Academy of Sciences, Leninsky Prospect 47, 119991 Moscow, Russia; 3 Laboratory of Synthetic Glycovaccines, N.D. Zelinsky Institute of Organic Chemistry, Russian Academy of Sciences, Leninsky Prospect 47, 119991 Moscow, Russia; 4 Laboratory of Metal-Complex and Nanoscale Catalysts, N.D. Zelinsky Institute of Organic Chemistry, Russian Academy of Sciences, Leninsky Prospect 47, 119991 Moscow, Russia; 5 Institute of Pharmacy, FSAEI HE I.M. Sechenov First MSMU of MOH of Russia (Sechenovskiy University), 119435 Moscow, Russia

**Keywords:** lysozyme, pheasant egg white, chitinase activity, fluorescence polarization, chitotriose hydrolysis, amino acid substitutions

## Abstract

Hen egg white lysozyme (HEWL) is a well-characterized antibacterial enzyme widely used in research and industry. However, little is known about the functional properties of lysozyme from other bird species, such as pheasants. This study aimed to compare the muramidase and chitinase activities of HEWL with the lysozyme from pheasant egg white (PEWL). Egg white samples were obtained from ten hens and ten pheasants. Lysozyme activity was assessed by two methods: a turbidimetric assay using *Micrococcus luteus* cell suspension to measure bacteriolytic (muramidase) activity and a fluorescence polarization assay (FPA) using FITC-labeled tri- and chitopentaoside substrates to measure the chitinase activity. The observed dissociation rate constant (k_obs_) was derived from the time-dependent FP signal. Turbidimetric measurements showed that PEWL possessed bacteriolytic activity approximately 30% lower than that of HEWL under the same conditions. However, FPA revealed a striking functional difference: whereas HEWL only bound the chitotrioside substrate without hydrolyzing it, PEWL was able to hydrolyze chitotriose. Both enzymes readily hydrolyzed the chitopentaoside substrate. The hydrolysis rate of the chitopentaoside was 50–70 times higher than that of the chitotrioside for PEWL. Sequence comparison identified six amino acid substitutions in PEWL (Phe3 → Tyr, His15 → Leu, Gln41 → His, Asn77 → His, Gln121 → Asn, Ile124 → Thr) relative to HEWL, which likely alter the conformation of subsites A–C and enable productive binding of the shorter oligosaccharide. Thus, despite high sequence similarity, pheasant lysozyme exhibits a unique functional property—the ability to hydrolyze chitotriose into N-acetylglucosamine. These findings expand functions of avian lysozymes and highlight the potential of alternative bird species as sources of enzymes with novel properties.

## 1. Introduction

Lysozyme (Lys) is a remarkable enzyme whose unique properties make it one of the key components of the innate immune system in humans and many animals [1,2,3]. Lys exerts potent antibacterial activity against Gram-positive bacteria by hydrolyzing the β-1,4-glycoside bond in the peptidoglycan layer of their cell wall [4,5,6]. However, the spectrum of Lys activity is considerably broader [1,4,7] and lysozyme is considered as an endogenous antibiotic [8].

Structurally, lysozyme is a small monomeric protein (14–15 kDa) stabilized by four disulphide bonds [9,10,11]. It is found not only in human tissues and secretions but also in vertebrates, invertebrates, bacteria and plants. Based on evolutionary criteria, lysozymes are divided into three families: chicken-type (c-type), goose-type (g-type) and invertebrate-type (i-type) [4]. Chicken (cLys, 129 aa, 14.3 kDa) and human (hLys, 130 aa, 14.7 kDa) lysozymes are both c-type and share 59% sequence identity; nevertheless, the antibacterial activity of hLys is three times higher than that of cLys.

The enzyme adopts a classic two-domain architecture (α-helical and β-domains, the latter containing a three-stranded antiparallel β-sheet) with a deep active-site cleft between them. The cleft is divided into six subsites (A–F) that interact with corresponding glycoside residues of the substrate [11]. Catalysis proceeds via an oxazolinium ion intermediate, with Glu35 acting as a proton donor and Asp52 stabilizing the transition state. Subsites A–C bind the non-reducing end of the oligosaccharide, while D–F bind the reducing end; the affinity for short oligomers (e.g., tri- and pentasaccharides) is largely governed by the architecture of subsites A–C, which directly affects hydrolysis kinetics.

The rise in bacterial resistance to conventional antibiotics drives the search for new antimicrobial agents and novel applications of known compounds such as lysozyme [12]. The clinical use of recombinant cell wall-degrading enzymes is therefore considered less risky in terms of resistance development [13]. For instance, lysozyme from various avian sources (quail, chicken, turkey) has been shown to inhibit HIV-1 at the stage preceding viral entry. Molecular modeling data suggest that lysozyme can bind to the CD4 receptor on target cells, thereby physically blocking virus attachment and entry [14]. Thus, lysozyme contributes to innate immunity not only as an antibacterial enzyme but also as an agent with direct antiviral activity, capable of interacting with nucleic acids and viral receptors [15].

Among avian lysozymes, the pheasant (*Phasianus colchicus*) egg white lysozyme (PEWL, specifically the PEL-1 isoform) is of particular interest. PEL-1 consists of 129 amino acid residues and contains 17 unique substitutions relative to hen egg white lysozyme (HEWL, identical to cLys). Several of these replacements lie within or near the active-site region and may significantly influence the orientation of catalytic residues, the geometry of the binding pocket, and consequently the rate of oligosaccharide hydrolysis. Comparative studies of HEWL and PEWL thus offer a unique opportunity to assess how active-center substitutions modulate enzymatic activity while preserving the overall protein fold. For kinetic characterization, chitotriose (trimer) and chitopentaose (pentamer) are selected as model substrates: chitopentaose occupies all six subsites and is cleaved between the fourth and fifth residues from the non-reducing end, whereas chitotriose binds only to subsites A–C. This difference allows the effects of substitutions in the A–C region to be distinguished from those in the D–F region.

Owing to its multifunctional properties, lysozyme has found diverse applications: as a pharmaceutical ingredient for treating infectious and inflammatory diseases of the oropharynx, oral cavity and gastrointestinal tract, as well as for wound and burn care [16]; as a natural preservative in food (e.g., cheese, meat, wine) to inhibit spoilage and pathogenic microorganisms [7,17]; and as an additive to skin-care products for its antibacterial and immune-modulating properties. Indeed, lysozyme is not merely a “natural antibiotic” but a versatile enzyme that provides comprehensive protection against bacteria, viruses and fungi while modulating the immune system and maintaining a balanced microbiota [18,19,20].

Avian eggs, especially chicken eggs, are an easily accessible source of lysozyme. Chicken egg white contains about 3.5% lysozyme relative to total protein, ensuring high yield, easy purification and consistent quality [9,21]. Consequently, HEWL has become the predominant form used in both scientific research and industrial applications [22]. Lysozymes from other bird species, such as pheasants, represent potential alternatives with distinct properties. Pheasants are increasingly bred in captivity for egg and meat production, and in this context lysozyme activity can serve as an important biochemical marker of bird health [23]. Reduced activity in eggs may indicate poor nutrition, stress or disease in the parent flock, and selective breeding could favor individuals with higher natural defenses (i.e., higher lysozyme activity), improving hatchability and chick viability [24]. Like many biochemical parameters, lysozyme activity can reflect environmental conditions and may be affected by pollutants such as heavy metals and pesticides; measuring activity in wild pheasants could thus provide an indirect assessment of ecosystem health. Furthermore, characterizing lysozymes from unconventional sources helps to link molecular structure to biological function and may lead to the discovery of enzymes with novel or unique properties.

## 2. Materials and Methods

### 2.1. Reagents and Materials

By conjugating fluorescein with chitotrioside and chitopentaoside [25,26], tracers **1a** and **2a** were produced (Figure 1). Eggs from ten hens and ten pheasants (one egg per bird) were purchased from a local market. Hen egg lysozyme (Sigma, Burlington, MA, USA), Na_2_HPO_4_, NaH_2_PO_4_, NaCl, KCl (Sigma-Aldrich, St. Louis, MO, USA), and lyophilized live bacterial cells *Micrococcus luteus* (Sigma-Aldrich, St. Louis, MO, USA) were the commercially available reagents utilized. FP-values were measured in a borosilicate glass tube (10 × 75 mm) with λex = 485 and λem = 535 nm using the portable Sentry-200 (Ellie LLC, Germantown, WI, USA). The collected data was processed using SigmaPlot 11 (Systat Software Inc., Palo Alto, CA, USA).

#### Pheasant Egg White Lysozyme

Pheasant egg white lysozyme (PEWL) was isolated from pheasant egg white according to a previously described procedure [27]. Briefly, pheasant egg white was diluted 10-fold with a carbonate buffer solution (20 mM sodium carbonate + 100 mM NaCl, pH 10.5) and then ultra-filtered through JetSpin^®^ 30 kDa MWCO filters (JetBiofil, Guangzhou, China) to collect the filtrate. The filtrate solution was dialyzed first against 20 mM KH_2_PO_4_ and then against deionized water. The final desalted solution was lyophilized and stored at −20 °C. MALDI-FT-ICR mass spectrometry analysis (Figure S1 in Supplementary Information) confirmed the correspondence of its amino acid sequence with that reported previously for PEWL (PDB entry 1GHL). The purity of the resulting product was evaluated using SDS-PAGE [28]; the purity of the product was 99% (Figure S2 in Supplementary Information). The concentration of lysozyme was determined by absorption at 280 nm using the extinction coefficient ε_280_^1%^ = 26.4.

### 2.2. Methods

#### 2.2.1. Fluorescence Polarization Analysis

Working solutions of fluorescein-labeled tri- or chitopentaoside tracers (TWS) were prepared in 50 mM phosphate buffer containing 137 mM NaCl, 2.7 mM KCl, and pH 7.0 (PBS). The tracer concentration was adjusted to 3 nM, which produced a fluorescence intensity of approximately 260,000 units. Then, 100 μL of egg white samples (pre-diluted 10-fold in the same buffer (50 mM phosphate, 0.11 M NaCl, pH 7.4)) were mixed with 900 μL of TWS. Following this, changes in the fluorescence polarization (FP) signal were recorded every 20 s over a ten-minute period. Each experiment was repeated at least three times. To determine the parameter k (the observed dissociation rate constant of the tracer from lysozyme, reflecting lysozyme activity), the measured time-dependent FP data for different lysozyme samples were fitted using the equation provided below:

(1) mP = mP_0_ + (mP_max_ − mP_0_) · e^−k^_obs_^*t^

where mP is the measured FP signal, mP_0_ is the polarization of free FITC-labeled tri- or chitopentaoside tracers, mP_max_ is the maximum FP signal achieved upon binding of the tracer and lysozyme, k_obs_ (the observed rate of decrease in FP) is an empirical parameter reflecting the combination of binding and hydrolysis, and t is a reaction time.

**Figure 1:**
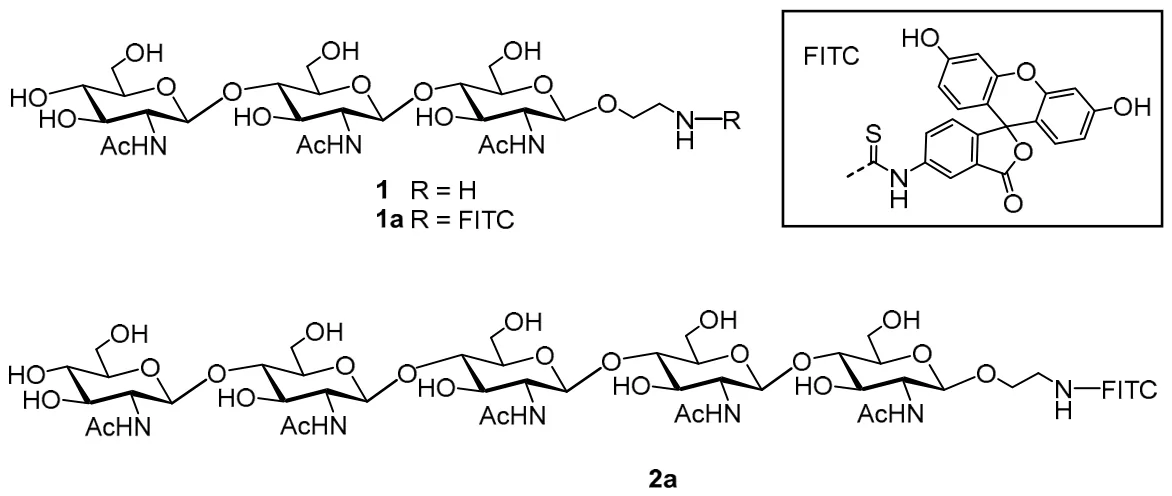
Structures of the synthetic oligosaccharide and FITC-labeled tracers used in this study.

#### 2.2.2. Apparent Pseudo-First-Order Hydrolysis Constants by FPA

Determination of apparent pseudo-first-order constants was determined using FPA. The depletion rates of fluorescein-labeled tri- or chitopentaoside tracers (final concentration (3 nM)) were studied under the influence of various concentrations of pure proteins HEWL and PEWL (7.5, 5.0, and 2.5 μM). Curve fitting was performed using the equation S = [S]_0_ exp(−k_1_ t) + C, where k_1_ is the apparent rate constant and C is the residual polarization after completion of the reaction. Fitting was performed using nonlinear regression (GraphPad Prism 6) with 95% confidence intervals.

#### 2.2.3. Active Lysozyme Determination by the Turbidimetry Method

Lysozyme activity was determined by monitoring the reduction in absorbance of a *Micrococcus luteus* cell suspension at 600 nm and 25 °C, with the resulting lysis rate serving as a measure of enzymatic activity [26]. The enzyme solutions were diluted and tested in a buffer composed of 10 mM Na_2_HPO_4_-NaH_2_PO_4_ and 150 mM NaCl at pH 7.0, which closely resembles the ionic environment of hen egg white. An initial absorbance of 0.70–0.72 was obtained by mixing 1 mL of the diluted lysozyme solution with 35 μL of a bacterial stock suspension containing 2 mg of the lyophilized *M. luteus* preparation. A calibration curve relating the rate of absorbance decrease to hen egg white lysozyme (HEWL) concentration showed linearity over the range of 0–5 μg/mL. All turbidimetric measurements were performed in a 1 mL quartz cuvette with a 10 mm light path using a Shimadzu UV-1801 spectrophotometer (Shimadzu, Kyoto, Japan).

#### 2.2.4. MALDI-FT-ICR-MS and NMR Control of Enzymatic Reaction of Chitotrioside 1 with PEWL

A solution of PEWL (0.53 mg, 35.3 nM) in PBS (150 µL) was added to a solution of chitotrioside **1** (1.18 mg, 1.76 µM) in PBS (150 µL). After being incubated at 37 °C overnight, the mixture was subjected to intermediate analysis by MALDI-FT-ICR mass spectrometry. High-resolution mass spectra were recorded on a solariX XR FT/ICR mass spectrometer equipped with a 15 T superconducting magnet, a ParaCell analyzer cell and a MALDI SmartBeam-II ion source (Bruker Daltonics, Bremen, Germany). The measurements were performed in positive (+) MS ion mode (HV Capillary: 4500 V; Spray Shield: −500 V) with a scan range of *m*/*z* 150–3000. External calibration of the mass spectrometer was performed using a low-concentration sodium trifluoroacetate solution (0.1 mg/mL in acetonitrile/water, 1:1). All MALDI-FT-ICR-MS measurements were recorded in positive ion mode with a grounded steel target plate (HCCA matrix, *m*/*z* 150–3000). All data visualization was processed using the Data Analysis 5.1 software package (Bruker Daltonics, Bremen, Germany).

Sample preparation for MALDI measurements: 0.5 μL of sample was mixed with 0.5 μL of HCCA-α-Cyano-4-hydroxycinnamic acid (saturated solution in 70/30 acetonitrile/water with 0.1% of TFA) using a pipet and spotted on a MALDI grounded steel target plate (Bruker Daltonics, Bremen, Germany) and allowed to dry at room temperature.

After 7 days of incubation at 37 °C, the reaction mixture was lyophilized, redissolved in D_2_O, and analyzed by NMR spectroscopy on a Bruker AV600 (600 MHz, Bruker Daltonics, Bremen, Germany) at 313K.

## 3. Results

### 3.1. Comparison of the Muramidase Activities of Chicken and Pheasant Lysozymes

Pheasant and chicken eggs were purchased from a local supermarket, and the samples of egg whites were collected. Then these samples were diluted 10-fold with PBS, and the activities of them were analyzed using a standard turbidimetric method. Notably, the 10-fold dilution in PBS was sufficient to minimize interference from other egg white proteins, such as ovomucoid and ovotransferrin, which are known to affect bacterial growth but do not directly contribute to lysozyme’s catalytic activity. Due to the unavailability of standard pheasant lysozyme, protein activity of egg white samples was estimated using a calibration obtained for HEWL (Helicon, Moscow, Russia). HEWL concentrations were taken from 0 to 6 μg/mL in increments of 1 μg/mL, and activities were measured using the turbidimetry. The principle of the turbidimetric assay consists of the decrease in absorbance of a *Micrococcus luteus* cell suspension. One unit of activity is defined as lysis of 0.6 μg of *M. luteus* in one minute, measured at wavelength λ = 600 nm at 25 °C. Then, the muramidase activity in collected egg white samples was studied. The results are presented in Figure 2.

These results revealed that both chicken and pheasant egg white samples exhibited lysozyme-dependent bacteriolytic activity. However, quantitative analysis indicated that the specific activity of pheasant lysozyme was 30% lower than that of chicken lysozyme under the same conditions.

### 3.2. Comparison of Chitinase Activities of Pheasant and Chicken Lysozymes Using the FPA Method

We have demonstrated the successful use of fluorescent substrates for the determination of lysozyme activity from different birds using the FPA method [25,26,29]. In this study, two lysozyme substrates [22], tri- **1a** and chitopentaoside **2a**, were used. As shown in previous studies, these substrates reacted differently with chicken lysozyme. When HEWL was added to the chitotrioside substrate, the FP signal increased sharply in a dose-dependent manner and did not change for an hour [22], and when added to the chitopentaoside substrate, as in the previous case, an initial sharp increase in FP was observed, followed by its decrease under the action of enzymatic hydrolysis for 10 min to the value of the free tracer.

When studying the chitinase activity of lysozyme from pheasant egg white (PEWL) samples from pheasant eggs, it turned out that in the case of the chitopentaoside substrate, the same picture was observed as for chicken lysozyme: an initial FP rises due to binding, followed by a time-dependent decrease as hydrolysis proceeded (Figure 3). This confirms that the chitopentaoside is a good substrate for both enzymes. However, unlike HEWL, pheasant lysozyme was able to hydrolyze the chitotrioside substrate. Specifically, addition of pheasant lysozyme to chitotriose **1a** resulted in a gradual decrease in the FP signal over time, indicating actual cleavage of the chitotrioside, whereas chicken lysozyme only bound the chitotrioside without hydrolysis (the FP signal remained elevated).

The kinetic parameters of chicken and pheasant lysozymes were compared. However, under our experimental conditions, the key assumptions of classical Michaelis–Menten kinetics were not satisfied, particularly the requirement that the substrate concentration be much higher than the enzyme concentration. Therefore, the Michaelis–Menten model was not appropriate for describing our system. To determine the kinetic parameters of our process, we used an alternative approach: substrate depletion kinetics analysis. Instead of measuring initial rates, the entire substrate consumption curve can be tracked. At low initial substrate concentration ([S]_0_), the reaction will follow pseudo-first-order kinetics, and information about the parameters can be calculated from this curve. For this purpose, the kinetic curves of the hydrolysis of chitotrioside **1a** and chitopentaoside **2a** by chicken and pheasant lysozymes were studied. Figure 4 shows the kinetic curves of the hydrolysis of chitopentaoside 1a (5 nM) at different concentrations of HEWL (Figure 4A). Linearized dependences in first-order coordinates are presented in Figure 4B, from which the apparent constant of substrate hydrolysis was calculated. Similar experiments were performed with chitotrioside **1a** and chitopentaoside **2a** using PEWL. The results are presented in Table 1. As can be seen from the presented data, the apparent pseudo-first-order hydrolysis constants for pure PEWL are higher than for HEWL.

To confirm the cleavage of the chitotriose moiety, synthetic chitotrioside **1** was incubated with PEWL and the reaction products were subsequently analyzed by mass spectrum entry and NMR spectroscopy. MALDI-FT-ICR mass spectrometry was employed for monitoring intermediate products in the reaction mixture, whereas NMR spectroscopy was used to identify the final products of enzymatic hydrolysis. Based on the combined spectroscopic data, the hydrolytic pathway shown in Figure 5 was proposed.

Synthetic chitotrioside **1** contains two inter-residue linkages, and mass spectrometric analysis indicated that both cleavage pathways occur with comparable probability. Signals corresponding to disaccharides **4** and **5** were detected in the mass spectra with similar intensities, together with signals of monosaccharides **3** and **6** (Figure S3 in Supplementary Information and Table 2).

Furthermore, NMR analysis of the reaction mixture after prolonged incubation detected only monosaccharides **3** and **6** without any sign of disaccharides **4** and **5** (Figure 6). These findings indicate that both spacered disaccharide **4** and chitobiose **5** are also susceptible to cleavage by PEWL. This remarkable observation distinguishes PEWL from HEWL, human lysozyme, and numerous other avian egg white lysozymes reported to date.

We then studied the hydrolysis rates of substrates **1a** and **2a** by PEWL diluted 10 times. As can be seen, the hydrolysis rate of the chitopentaoside substrate is 50–70 times higher than that of the chitotrioside substrate (Table 3). Perhaps a slight difference in the active site of pleasant lysozyme allows it to hydrolyze a substrate with fewer sugars.

## 4. Discussion

The fluorescence polarization (FP) methods are increasingly used today for infectious diagnostics and analysis of biological recognition processes [30]. In our work using the FPA method with fluorescently labeled oligosaccharides of different lengths as probes, we found that lysozymes from chicken and pheasant egg proteins exhibit different activities towards trisaccharide.

The interaction of chicken lysozyme with oligosaccharides of various lengths has been well studied [31,32]. Compared with classical methods such as turbidimetry and chromatography, fluorescence polarization assay (FPA) enables real-time monitoring of changes in ligand binding and hydrolysis within a single measurement procedure. This is particularly useful for lysozyme, which can exhibit both catalytic and lectin-like binding activities.

The active site of chicken lysozyme is an elongated cleft with six subsites, designated A through F. For efficient hydrolysis, lysozyme requires at least 4–6 saccharide units for proper positioning of the substrate in the active site (structural data for the hexasaccharide–lysozyme complex PDB 1LZ1). Catalytic hydrolysis of the glycosidic bond occurs specifically between the D and E subsites. Based on this model, two types of enzyme–substrate complexes are distinguished: (1) a non-productive complex, where the oligosaccharide occupies only subsites A–C and does not undergo hydrolysis, and (2) a productive complex, where the substrate reaches subsites D–F, enabling correct positioning for catalysis. Accordingly, chitotriose (GlcNAc_3_) binds exclusively at A–C and is not hydrolyzed, whereas chitopentaose (GlcNAc_5_) and chitohexaose (GlcNAc_6_) can form productive complexes. The higher catalytic efficiency toward the hexasaccharide compared to the pentasaccharide is attributed to additional stabilization provided by subsite F. This is a well-established model [31,32]. provides a structural basis for understanding the substrate-length dependence of lysozyme activity.

In our previous study, using FPA with fluorescein-labeled tri- and chitopentaosides, we demonstrated [29] that chitinase activity is not detectable in the egg whites of duck, goose, turkey, peacock, and ostrich. Since duck, turkey, and peacock lysozymes are C-type, while goose and ostrich lysozymes are G-type, it was hypothesized that the primary reason for the lack of activity in these tests is due to structural differences between lysozyme isoforms. Although C-type lysozymes from egg whites of different birds retain high amino acid sequence homology, and their active sites contain the same set of amino acids, even minor differences in amino acid sequence can affect binding and catalytic efficiency toward chitooligosaccharides. It was also shown that lysozyme isolated from pheasant egg white exhibited hydrolytic activity towards glycol-chitin and chitooligosaccharides with a chain length of 5 and 6 monomers ((GlcNAc)_5_ and (GlcNAc)_6_) [33]. In this case, the enzyme hydrolyzes the (GlcNAc)_6_ molecule, predominantly breaking the fourth glycosidic bond, counting from the non-reducing end of the chain.

Pheasant lysozyme is a classic representative of the c-type (chicken-type). Amino acid sequence studies have shown that pheasant lysozyme is very similar to chicken lysozyme (differing in only a few amino acids [34,35]). The pheasant lysozyme proved to consist of 129 amino acid residues, and a molecular weight of 14,423 Da was calculated. This lysozyme had 6 amino acid substitutions when compared with hen egg white lysozyme: Phe3 to Tyr, His15 to Leu, Gln41 to His, Asn77 to His, Gln 121 to Asn, Ile124 to Thr [34]. This allows us to assume with a high degree of probability that its catalytic properties, including chitinase activity, are qualitatively similar to chicken lysozyme.

Our results reveal both quantitative and qualitative functional differences between pheasant and chicken lysozymes, which can be attributed to the six amino acid substitutions. This striking functional difference suggests that the six amino acid substitutions, particularly those near the active site (e.g., His15 → Leu, Gln41 → His, Asn77 → His, and Ile124 → Thr), may alter the conformation of subsites A–C, allowing the chitotrioside to be positioned catalytically rather than merely inhibited.

The replacement of His15 with Leu removes a polar side chain that normally interacts with the N-acetyl group of the sugar at subsite C, potentially reducing steric hindrance for the shorter chitotrioside. Likewise, the Gln41 → His and Asn77 → His substitutions introduce additional histidine residues that might facilitate acid-base catalysis or alter water-mediated hydrogen bonding networks. The Ile124 → Thr substitution near the C-terminal domain could also affect the flexibility of the active site cleft. These structural changes, though subtle, appear to relax the strict length requirement for substrate binding, enabling hydrolysis of chitotriose.

The chitinase-related side activity of pheasant lysozyme, particularly toward short oligosaccharides such as chitotriose, warrants consideration in the context of its ground-nesting ecology. While lysozymes typically exhibit 2–3 orders of magnitude lower catalytic efficiency toward chitin than toward bacterial peptidoglycan, even marginal hydrolysis could contribute to slowing fungal spore germination on the eggshell surface under the humid microclimate of a ground nest. Importantly, substitutions such as Asn77 → His introduce a positive charge near the substrate-binding cleft—a feature reminiscent of true chitinases (GH18)—and likely subtly alter affinity for oligosaccharide-containing substrates. However, this remains a structural prediction requiring experimental validation via site-directed mutagenesis and kinetic assays.

## 5. Conclusions

Thus, this work demonstrates that pheasant egg white lysozyme, despite being highly homologous to chicken lysozyme, possesses a unique functional property—the ability to hydrolyze chitotriose. This distinctive property of PEWL may find applications in biotechnology where anti-chitin activity is required (e.g., for combating fungal infections), as an alternative source of lysozyme for food production, and as a model for studying the substrate specificity of enzymes. Furthermore, studying lysozyme variants from different bird species may reveal additional examples of divergence in substrate specificity.

## Figures and Tables

**Figure 2:**
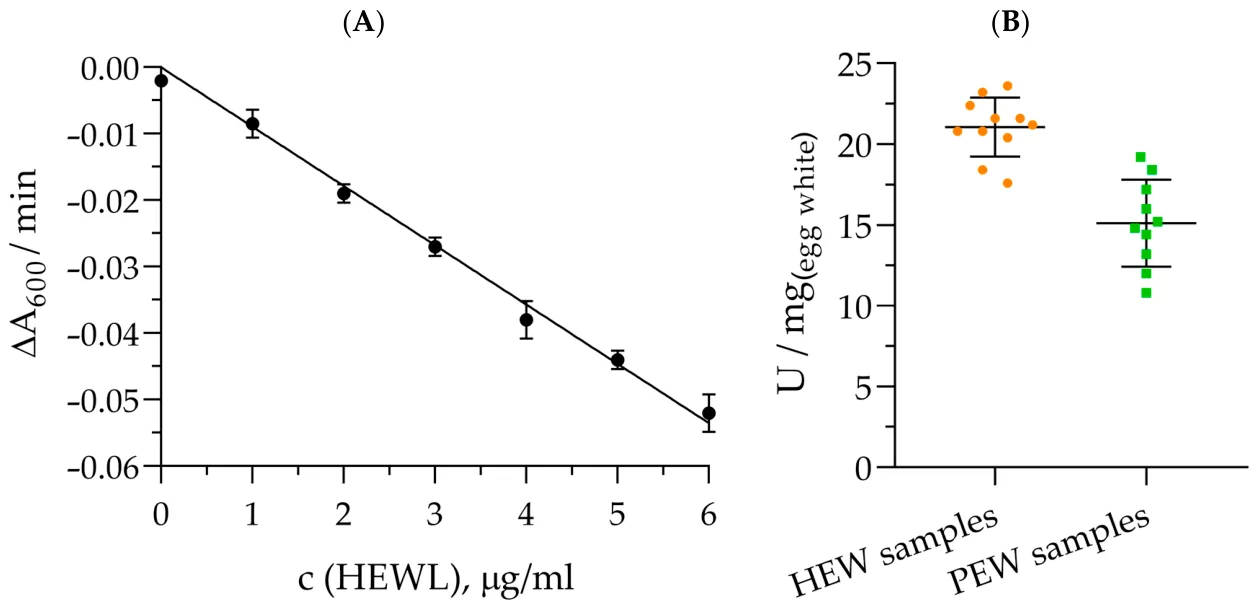
(**A**) The calibration curve of the activity of HEWL by the turbidimetric method. (**B**) Comparison of lysozyme activity in samples obtained from chicken and pheasant egg whites using turbidimetry (n = 3).

**Figure 3:**
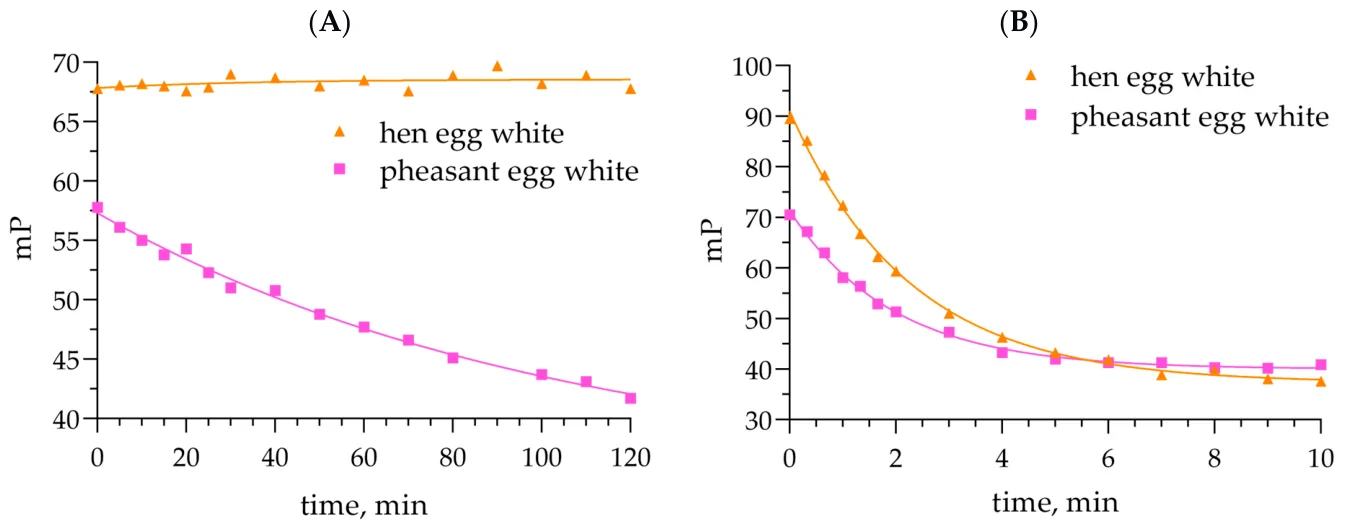
Comparison of hydrolysis of chitotrioside **1a** (**A**) and chitopentaoside **2a** (**B**) substrates by pheasant and chicken lysozymes (final enzyme concentration 2.7 µM).

**Figure 4:**
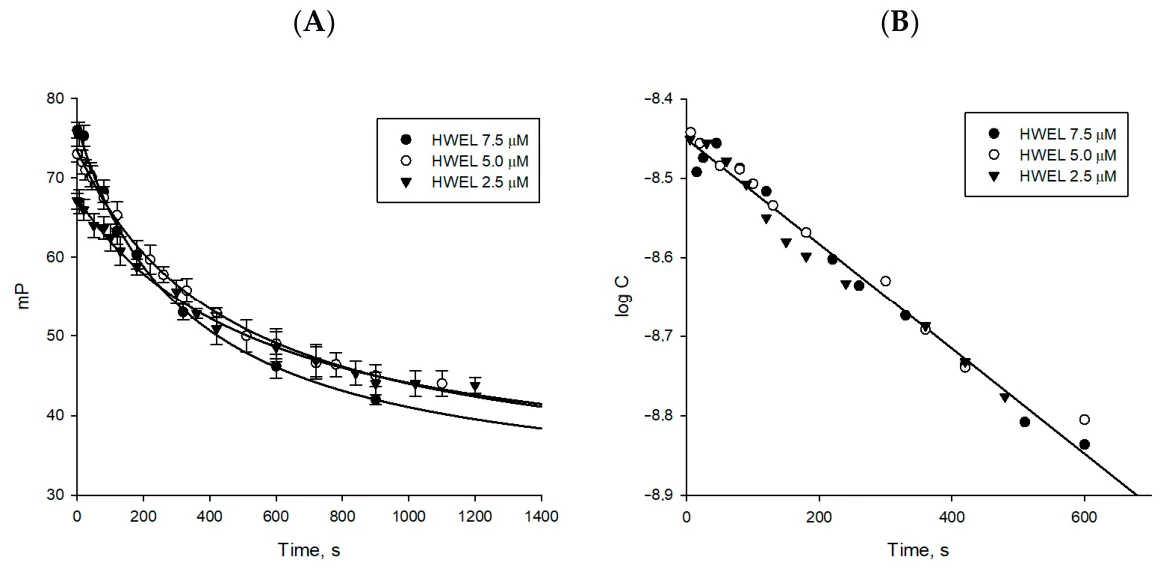
(**A**) Hydrolysis curves of chitopentaoside **2a** (3 nM) at different concentrations of HEWL (n = 3). (**B**) Linearized hydrolysis curves in first-order coordinates (n = 2).

**Figure 5:**
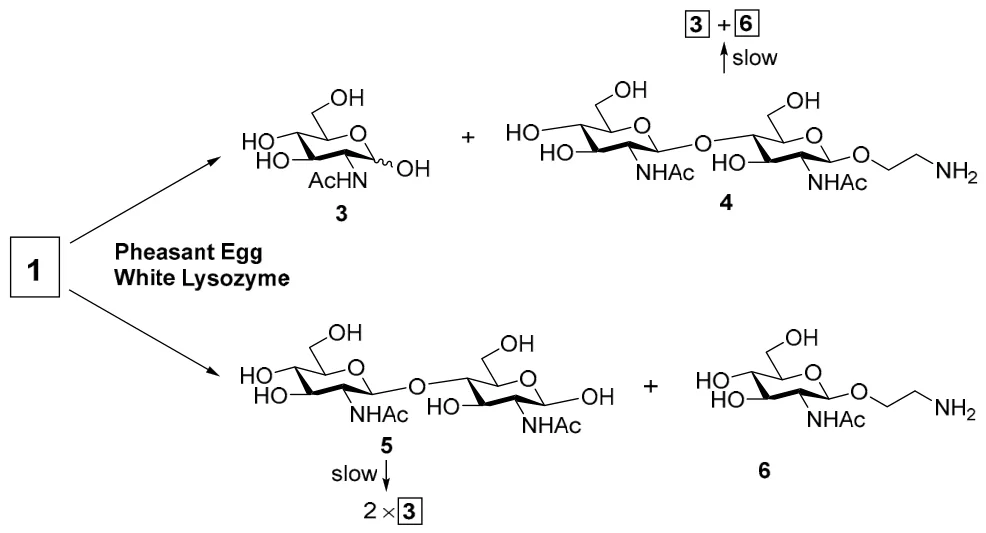
Proposed hydrolytic pathway of synthetic chitotrioside **1** catalyzed by PEWL.

**Figure 6:**
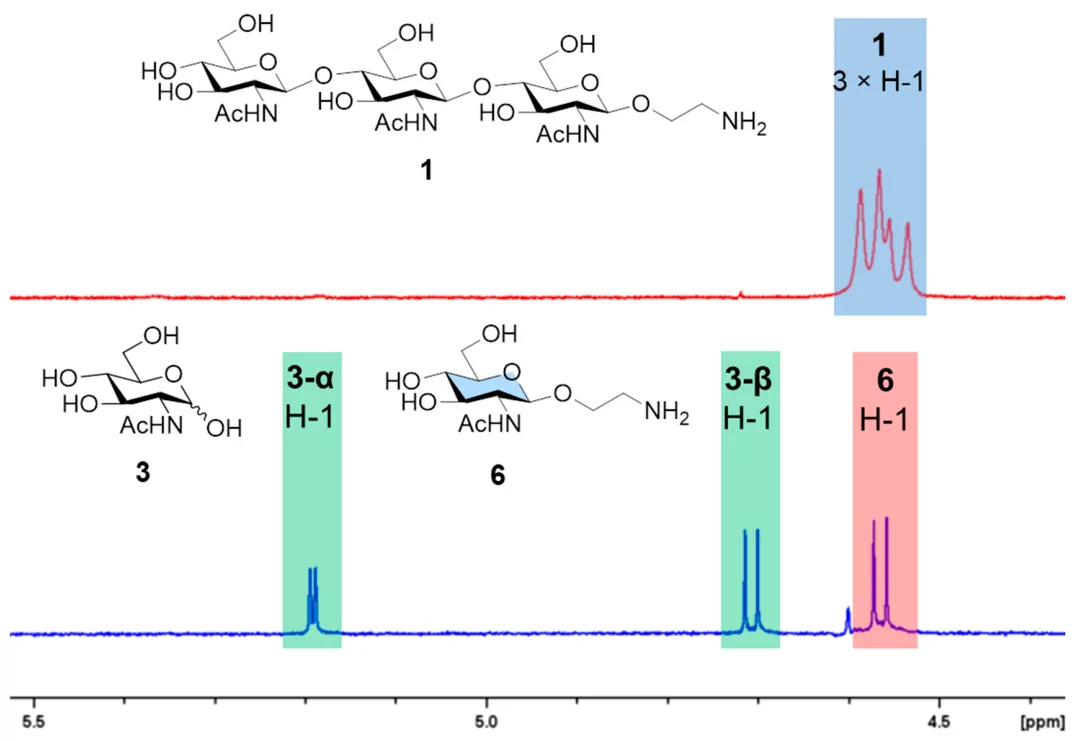
Anomeric region of ^1^H NMR spectrum of starting **1** (**top**) and the final products of **1** digestion with PEWL (**bottom**).

**Table 1:** Apparent pseudo-first-order hydrolysis constants of substrates chitotrioside **1a** and chitopentaoside **2a** by HEWL and PEWL.

Substrate	Apparent Pseudo-First-Order Hydrolysis Constants, min^−1^	
	HEWL	PEWL
Chitotrioside **1a**	-	0.006 ± 0.002
Chitopentaoside **2a**	0.040 ± 0.012	0.450 ± 0.025

**Table 2:** Theoretical and found molecular ions *m/z* of the oligosaccharide fragments obtained after cleavage of chitotrioside **1** by PEWL.

Compound	Chemical Formula	Calc.	Found
**1**	C_26_H_46_N_4_O_16_	693.28010 [M + Na]^+^	693.27878
**3**	C_8_H_15_NO_6_	244.07916 [M + Na]^+^	244.07899
**4**	C_18_H_33_N_3_O_11_	490.20073 [M + Na]^+^	490.20005
**5**	C_16_H_28_N_2_O_11_	447.15853 [M + Na]^+^	447.15805
**6**	C_10_H_20_N_2_O_6_	287.12136 [M + Na]^+^	287.12114

**Table 3:** Results of hydrolysis of tri- and chitopentaoside substrates with pheasant lysozyme using the FPA method.

Egg White Sample	k_obs_ (1a), min^−1^	k_obs_ (2a), min^−1^	Ratio k_obs_ (2a)/k_obs_ (1a)
1	0.0093	0.516	55
2	0.0136	0.672	49
3	0.0049	0.246	50
4	0.0066	0.504	76
5	0.0069	0.510	74
6	0.0059	0.318	54
7	0.0096	0.486	51
8	0.0047	0.300	64
#9	0.0040	0.270	68
#10	0.0038	0.288	76

## Data Availability

The original contributions presented in this study are included in the article/Supplementary Materials. Further inquiries can be directed to the corresponding authors.

## Supplementary Materials

The following supporting information can be downloaded at: https://www.mdpi.com/article/10.3390/foods15193402/s1, Figure S1: MALDI-FT-ICR mass spectrometry analysis of pheasant egg white lysozyme (PEWL); Figure S2: SDS-PAGE analysis of pheasant egg white lysozyme (PEWL); Figure S3: MALDI-FT-ICR mass spectrometric analysis of the reaction mixture obtained by cleavage of trisaccharide **1** by PEWL.

## Abbreviations

The following abbreviations are used in this manuscript:

FPA	fluorescence polarization analysis
FP	fluorescence polarization
HEWL	hen egg white lysozyme
hLys	human lysozyme
Lys	lysozyme
PBS	phosphate-buffered saline
PEWL	pheasant egg white lysozyme
TWS	tracer working solution
