# Supplementary material for: Pheasant Egg White Lysozyme vs. Hen Egg White Lysozyme: A Comparative Study of Enzymatic Activity
Source: Foods. 2026 Sep 24;15(19):3402. doi: 10.3390/foods15193402 (PMC13664433; doi:10.3390/foods15193402)
Supplement: Supplementary file 1 [file foods-15-03402-s001.zip › foods-4466311-supplementary.pdf]

Supplementary Information

Pheasant Egg White Lysozyme *vs.* Hen Egg White Lysozyme: A Comparative Study of Enzymatic Activity

Liliya I. Mukhametova, Dmitry O. Zherdev, Anton N. Kuznetsov, Julia V. Burykina, Olga N. Yudina, Svetlana M. Filimonova, Sergei A. Eremin, Vadim B. Krylov*, Nikolay E. Nifantiev*

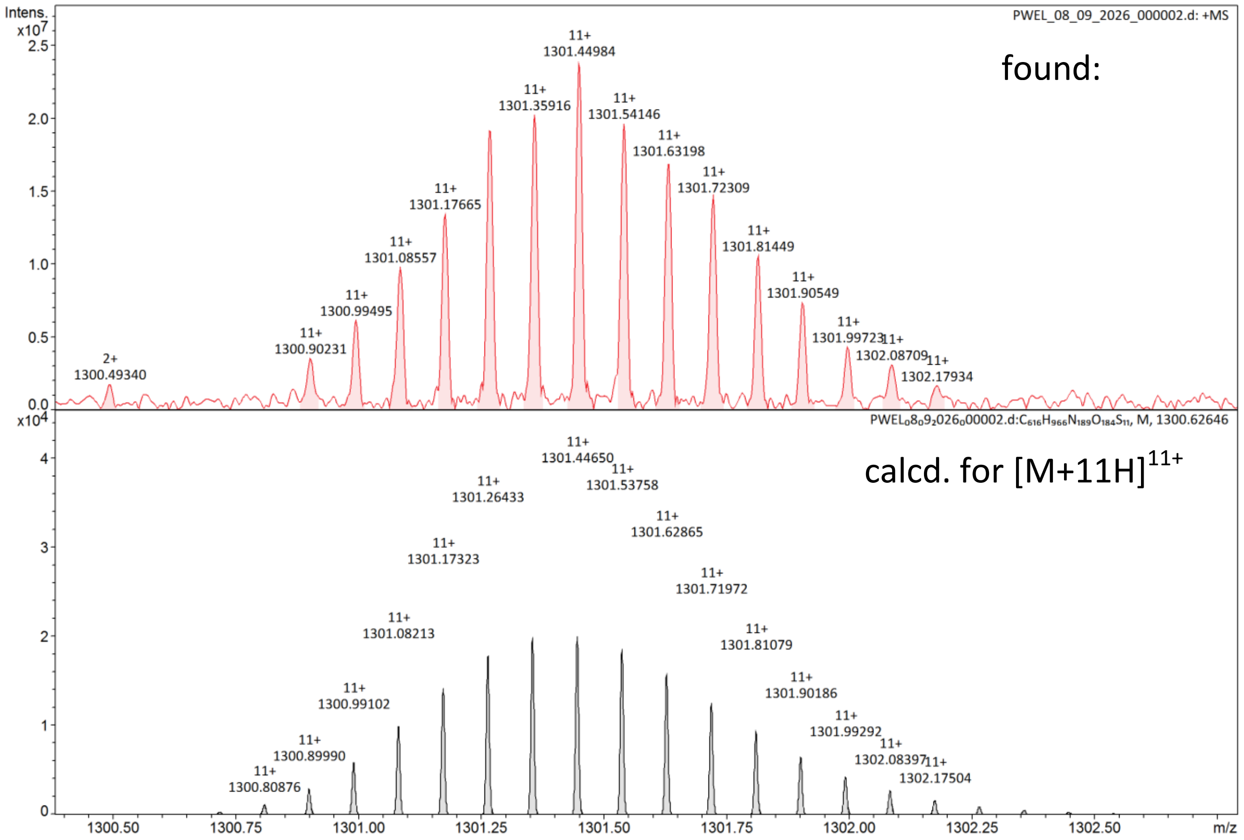

Figure S1. MALDI-FT-ICR mass spectrometry analysis of pheasant egg white lysozyme (PEWL). Top: experimentally obtained mass spectrum. Bottom: simulated mass spectrum based on the PEWL sequence (GKVYGRCELAAAMKRMGLDNYRGYSLGNWVCAAKFESNFNTGATNRNTDGSTDYGILQINSRWWCNDGRTPGSKNLCHIPCSALLSSDITASVNCAKKIVSDGNGMNAWVAWRKHCKGTDVNVWIRGCRL) from PDB entry **1GHL**. Brutto formula: $C_{616}H_{955}N_{189}O_{184}S_{11}$.

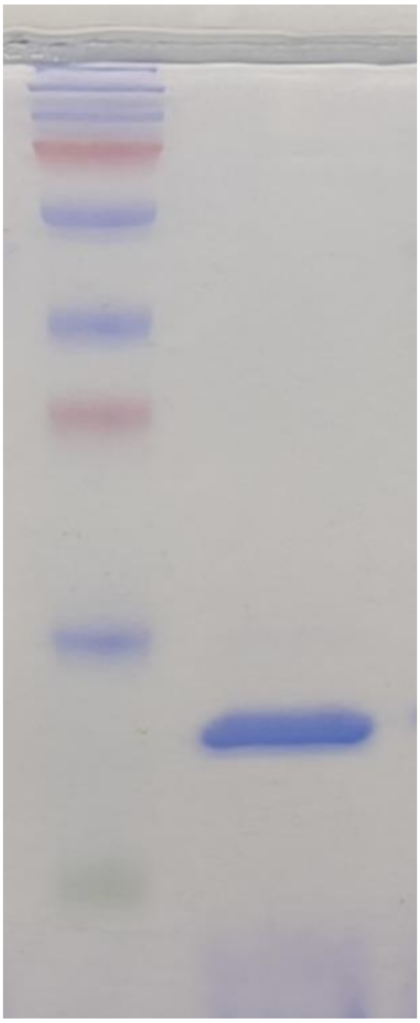

Figure S2. SDS-PAGE analysis of pheasant egg white lysozyme (PEWL). Left lane: molecular weight markers (10, 15, 25, 35, 55, and 70 kDa). Right lane: PEWL (theoretical molecular weight, 14.3 kDa).

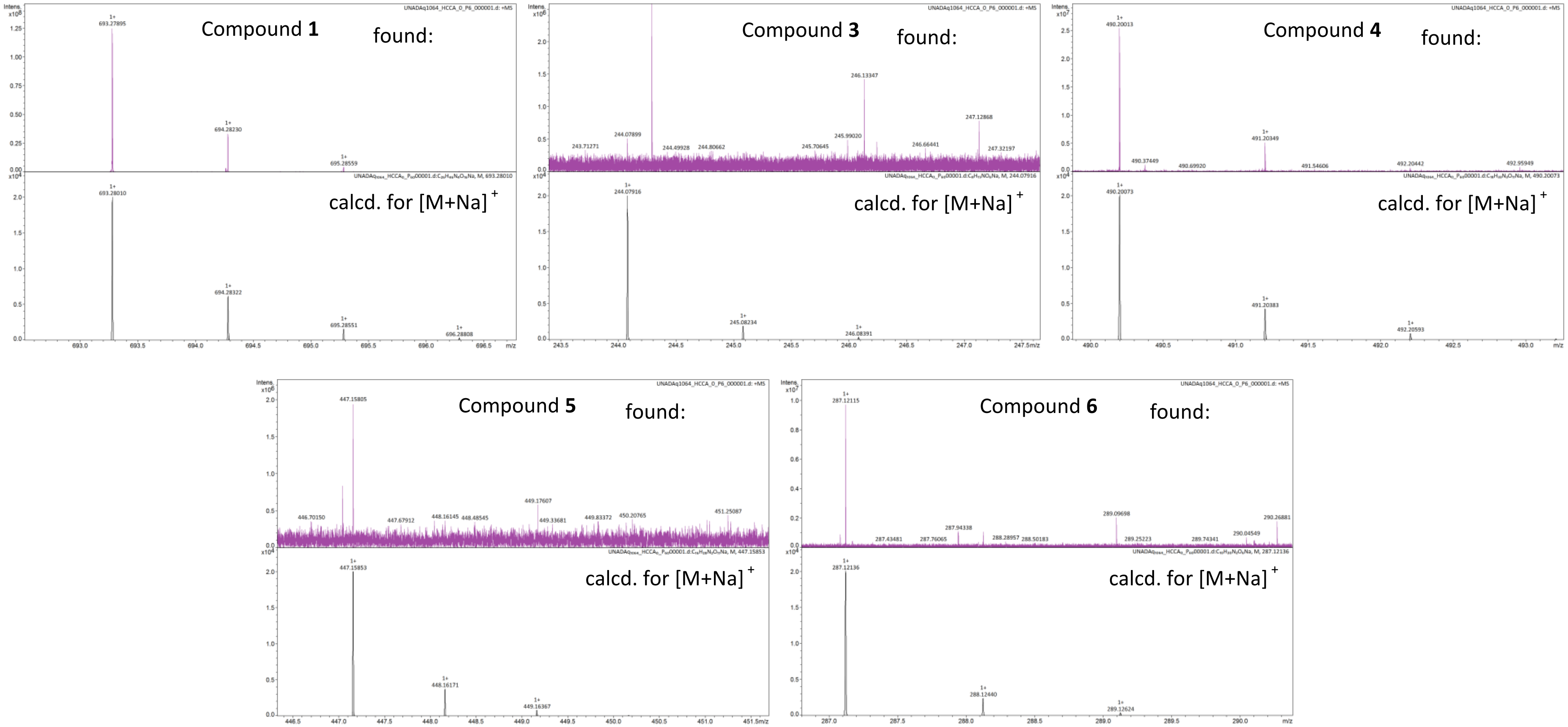

Figure S3. MALDI-FT-ICR mass spectrometric analysis of the reaction mixture obtained by cleavage of trisaccharide **1** by PEWL.
